# Role of P2X7 receptor in the progression and clinicopathological characteristics of gastric cancer

**DOI:** 10.1038/s41598-024-81515-7

**Published:** 2024-12-30

**Authors:** Wen-jun Zhang, Xiong-wei Pi, Yi-guan Le, Teng-zheng Li

**Affiliations:** 1 Department of Rehabilitation Medicine, The Second Affiliated Hospital, Jiangxi Medical College, Nanchang University, Nanchang city, Jiangxi province, China; 2 Gastrointestinal Surgery, The Second Affiliated Hospital, Jiangxi Medical College, Nanchang University, Nanchang city, Jiangxi province, China; 3 Department of Gastroenterology, The Second Affiliated Hospital, Jiangxi Medical College, Nanchang University, Nanchang city, Jiangxi province, China

**Keywords:** Gastric cancer (GC), P2X7 receptor (P2X7R), Clinicopathological features, Proliferation, Migration and invasion, Cancer, Cell biology

## Abstract

P2X7 receptor (P2X7R) plays a role in regulating tumor progression, but it is unclear whether P2X7R affects the pathological characteristics of patients with gastric cancer and the activity of gastric cancer cells. Therefore, this study preliminarily investigated the relationship between P2X7R and clinicopathological features of patients with gastric cancer, and further explored the effect of P2X7R on the proliferation, migration and invasion of gastric cancer cells through functional experiments. The results showed that P2X7R was highly expressed in gastric cancer tissues and gastric cancer cells. High expression of P2X7R was closely related to lymphatic metastasis, vascular invasion and Tumor-Node-Metastasis (TNM) stage in patients with gastric cancer. High expression of P2X7R predicted poor overall survival in patients. Moreover, the activation of P2X7R by ATP and its analogue BzATP increased the calcium current of gastric cancer cells, enhanced YF actin stress and cell viability, and promoted the proliferation, migration and invasion of gastric cancer cells. While P2X7R antagonists (A438079 and AZD9056) inhibited the proliferation, migration and invasion of gastric cancer cells induced by ATP. Therefore, the data obtained in this experiment suggest that P2X7R may be another potential molecular target for the prevention and treatment of gastric cancer.

## Introduction

Gastric cancer (GC) is a common malignant tumor in the digestive system, and its pathological mechanism is complex. Its prevention and treatment have always been an important topic to be solved urgently. Although a variety of combined methods can be used to control the progression of GC, such as surgical resection, targeted therapy, immunotherapy, radiotherapy and chemotherapy. The combination of these methods can improve the quality of life and survival of patients with GC. However, the growth and metastasis of GC are the key to the failure of treatment^1,2^. Therefore, it is of great significance for the treatment of GC to explore new molecular targets related to the progression of GC and to make favorable prediction.

The structure of P2X7 receptor (P2X7R) protein includes three subunits, namely intracellular domain (a N-terminus and a long cytoplasmic C-terminus), two transmembrane domains (TM1 and TM2) and extracellular domain (containing ATP-binding sites), and is expressed in almost all human tissues. P2X7R is activated by binding to ATP and is involved in regulating the progression of most diseases, including inflammation, pain, and tumor^3,4^. At present, different studies have revealed that P2X7R is expressed in most tumor tissues and tumor cells, such as glioma^5^, bladder cancer^6^, breast cancer^7^, colorectal cancer^8^, melanoma^9^. Acute myeloid leukemia^10^, and osteosarcoma^11,12^, which is involved in the regulation of tumor progression. After tumorigenesis, tumor cells and non-tumor cells can release a large amount of ATP into extracellular, bind to P2X7R, further open the ion channel on the membrane (mainly mediates calcium influx), and regulate the biological activity of tumor cells^13,14^. Generally speaking, P2X7R activation promotes the growth, migration and metastasis of tumors, while its antagonists such as A438079, BBG or A740003 have pharmacological properties of inhibiting the progression of tumors^15,16^. Activation of P2X7R by ATP or BzATP can promote EMT, migration and invasion in non-small cell lung cancer A549 cells, but this effect is attenuated by treatment with P2X7R antagonist A438079^17^. ATP or BzATP induce the activation of P2X7R, which promotes the proliferation, migration and invasion of colorectal cancer cells. On the contrary, knocking down the expression of P2X7R or using its antagonists A438079 or AZD9056 can inhibit the proliferation and migration of colorectal cancer cells^18^. Studies have shown that P2X7R is highly expressed in human breast cancer cells, and its activation promotes the migration, expansion and invasion of tumor cells by enhancing the SK3 channel and cysteine cathepsin^19^. In vivo experiments also showed that P2X7R activation promoted the growth of tumor^20^.

All these studies reveal the fact that P2X7R plays an important regulatory role in tumor progression. However, little is known about the inherent relationship between P2X7R and GC. Therefore, this study explored the relationship between P2X7R and clinicopathological features of patients with GC and its effect on the biological behavior of GC cells, revealing that P2X7R may be used as another important molecular target for GC prediction and treatment.

## Methods and materials

### GC patient data and tissue samples

The studies involving human participants was approved by the Ethics Committee of the Second Affiliated Hospital of Nanchang University, and informed consent was obtained from every participant. All protocols were approved by the Ethics Committee, China. All methods were performed in accordance with the relevant guidelines and regulations. According to the criteria of the National Comprehensive Cancer Network classification system, we collected 86 (54 male, 32 female) surgically removed GC tissues and adjacent tissue samples (at least 5 cm away from the tumor tissue). These specimens were from the Second Affiliated Hospital of Nanchang University from 2018 to 2019, and the patients did not undergo radiation and chemotherapy treatment before surgery. The clinical characteristics and pathological data of these patients were from the medical record and pathology report of our hospital. Histological types were assigned according to the criteria of the National Comprehensive Cancer Network classification system. TNM stages of these cases were specified according to the American Joint Committee on Cancer (AJCC), 7th edition. Hematoxylin–eosin staining was used to assess tumor vascular invasion. Survival data of the patients were collected from hospital medical data-processing record. The overall survival was determined as the interval between the day of surgery and the day of death.

### Immunohistochemistry and H-E staining

GC tissues and adjacent non-tumor tissues were embedded in paraffin and sectioned, and then deparaffinized and rehydrated. Slices were placed into the boiled antigen retrieval solution for 5 min, and then were washed 3 times with PBS. 3% hydrogen peroxide was added and incubated for 15 min, and then was washed with PBS. 200 μl of 5% goat serum was added and incubated for 30 min without washing. 200 μl rabbit P2X7R primary antibody (1:200, Sanying Biotechnology Co., Ltd., Wuhan, China) and incubated overnight at 4 °C. Next day, tissues were washed 3 times with PBS, goat anti-rabbit secondary antibody (1:500, Sanying Biotechnology Co., Ltd., Wuhan, China) was added and incubated for 1 h, and then washed with PBS. DAB chromogenic solution was added to react for 2–5 min, after the tissues were dyed brownish yellow, and washed with tap water. Subsequently, hematoxylin was added to stain for 1–2 min, and was rinsed with tap water. Then, dehydrated, transparent and sealed. Observed and photographed under an inverted microscope. Analyzed the distribution and number of P2X7R positive cells.

H-E staining: Tissue sections were processed according to the above immunofluorescence process, and then, hematoxylin was added to stain the nuclei for 5 min, and rinsed with tap water. Eosin was added and stained for 1 min, then rinsed with tap water. Tissue sections were then dehydrated, transparent, and sealed. Observed under an inverted microscope and taken photos.

### P2X7R expression score

The expression of P2X7R in tumor tissues was evaluated by IHC-score, which was obtained multiplying the staining intensity (0: negative, 1: weak staining, 2: moderate staining, 3: strong staining) by the distributions (0, < 10%; 1, 10–25%; 2, 25–70%; 3, > 70%) and ranged from 0 to 9. The median value of the IHC score was considered as the cutoff criterion. In the 86 patient specimens. IHC score below the criterion was considered as the low P2X7R group and IHC score above the criterion was considered as the high P2X7R group.

### Cell culture

7901 and 803 cell lines (Cell Bank of the Chinese Academy of Sciences, Shanghai, China) were cultured with 1640 medium containing 10% FBS (Doctor DE Biological, Wuhan, China). Cell culture medium was exchanged every two to three days, and the cell growth condition was observed under a microscope.

### Western-blotting

Total proteins were extracted from 7901 and 803 cells, these cells were treated/untreated with ATP (200 μM), BzATP (10 μM), and ATP + A438079 (10 μM). Protein loading (10 μl per sample), electrophoresis, membrane transfer, and fluorescence reactions were performed according to the experimental procedures. The primary antibodies were used for the target gene were all rabbit polyclonal antibodies, and the dilution ratios were as follows: P2X7R (1:1000, Sanying Biotechnology Co., Ltd., Wuhan, China) or β-actin (1:1000, Doctor DE Biological, Wuhan, China) for overnight at 4 °C. The goat anti-rabbit secondary antibody was 1:5000 (Boster Biotechnology Co., Ltd, Wuhan, China). β-actin was used as an internal reference to standardize the target gene.

### YF actin staining experiment

1 × 10^4^ 803 or 7901 cells were cultured in 24-well plates, and were treated with ATP (200 μM), ATP + A438079 (10 μM) and ATP + AZD9056 (10 μM) for 24 h. The medium from the 24-well plate was removed and washed with PBS for 3 times. 400 μl 4% paraformaldehyde was added to each hole and were fixed on ice for 15 min. 400 μl 0.5%Trion X-100 was added to each hole, and acted for 30 min, and was washed with PBS. 400 μl PBS diluted 3 μl YF fluorescence labelled ghost pen cyclic peptide solution was added to each well and incubated for 40 min. Then the morphological changes of cells and actin filaments were observed under fluorescence confocal microscope.

### Fluo-4AM assay

Fluorescence indicator Fluo-4AM (Sanying Biotechnology Co., Ltd., Wuhan, China) was used to measure the change of free internal calcium concentration. The 24-well culture plate was taken out, and cells were washed 3 times with PBS. 300 µl 4 µM Fluo-4AM working solution was added and incubated at 37 °C for 1 h, and then were washed 3 times with PBS. Then, cells were incubated for an additional 10 min in PBS. Fluo-4 fluorescence signals were detected from 5 cells using a confocal microscope (Danmic Global, LLC, San Jose, CA, USA), and the average Fluo-4 fluorescence signals was obtained from three samples separate experiments.

### Cell scratch assay

A Marker pen was used to draw 6 horizontal lines behind each 6-well plate. The horizontal lines passed through each hole at 1 cm intervals. Cell suspensions were added to each well (approximately 7 × 10^5^ cells) and placed the 6-well plate into CO_2_ incubator for cultivation. After observed that the cells covered the entire hole, scratches were made with the sterile 10 µl gun head, and then the cells were washed with PBS. 1640 medium containing 10% FBS was added and putted them in a CO_2_ incubator for cultivation. Cells were treated with or without ATP (200 μM), ATP (300 μM), BzATP (10 μM), A438079 (10 μM), or ATP + A438019 (10 μM), observed and photographed under an inverted microscope at 0 h and 24 h, respectively, and the percentage of wound healing between cell scratches was calculated.

### Cell migration and invasion experiment

Analysis of GC cells invasion and migration abilities by using 24-well Transwell chamber (Doctor DE Biological, Wuhan, China). The filter of the upper insert was coated with Matrigel **(**Migration assay omitted) before used and planted/ 200 µl serum-free cell suspension (2 × 10^4^ cells), and the lower insert was added with 200 μl of 1640 medium containing 10% FBS. After cells were treated with or without ATP (200 μM), ATP (300 μM), BzATP (10 μM), A438079 (10 μM), or ATP + A436079 (10 μM) for 24 h, and the invaded and migrated cells were fixed with 4% paraformaldehyde for 30 min. Subsequently, the cells were stained with crystal violet, and the cells were counted in 5 random fields under the microscope.

### EDU proliferation and survival assay

7901 and 803 cells were inoculated into 24-well plates in the presence or absence of ATP (200 µM), ATP (300 µM), BzATP (10 µM), ATP + A438079 (10 µM), or A438079 (10 µM) for 24 h. Next, 100 μl of 10 μM EDU working solution was added and incubated for 2 h. After incubation, cells were fixed with 4% paraformaldehyde for 15 min. Next, 50 µl of 2 mg/ml glycine solution was added for 5 min, and then 0.1% TriX-100 (diluted in PBS) was added and incubated for 15 min. Subsequently, Apollo and Hoechst staining solutions were added. Cells were observed and photographed by using an inverted fluorescence microscope.

### Statistical method

The P2X7R expression and clinicopathological features were analyzed using the Pearson χ^2^ test. In the cell line studies, the data was presented as mean ± SEM. Test of significance was done with Student *t* test. Alternatively, when the homogeneity test of variance fails, Mann–Whitney rank sum test was used. Kaplan–Meier curves was constructed by GraphPad program. *P* values of less than 0.05 were considered to have significant statistical significance.

## Results

### Upregulation of P2X7R expression in GC tissues

**Fig. 1:**
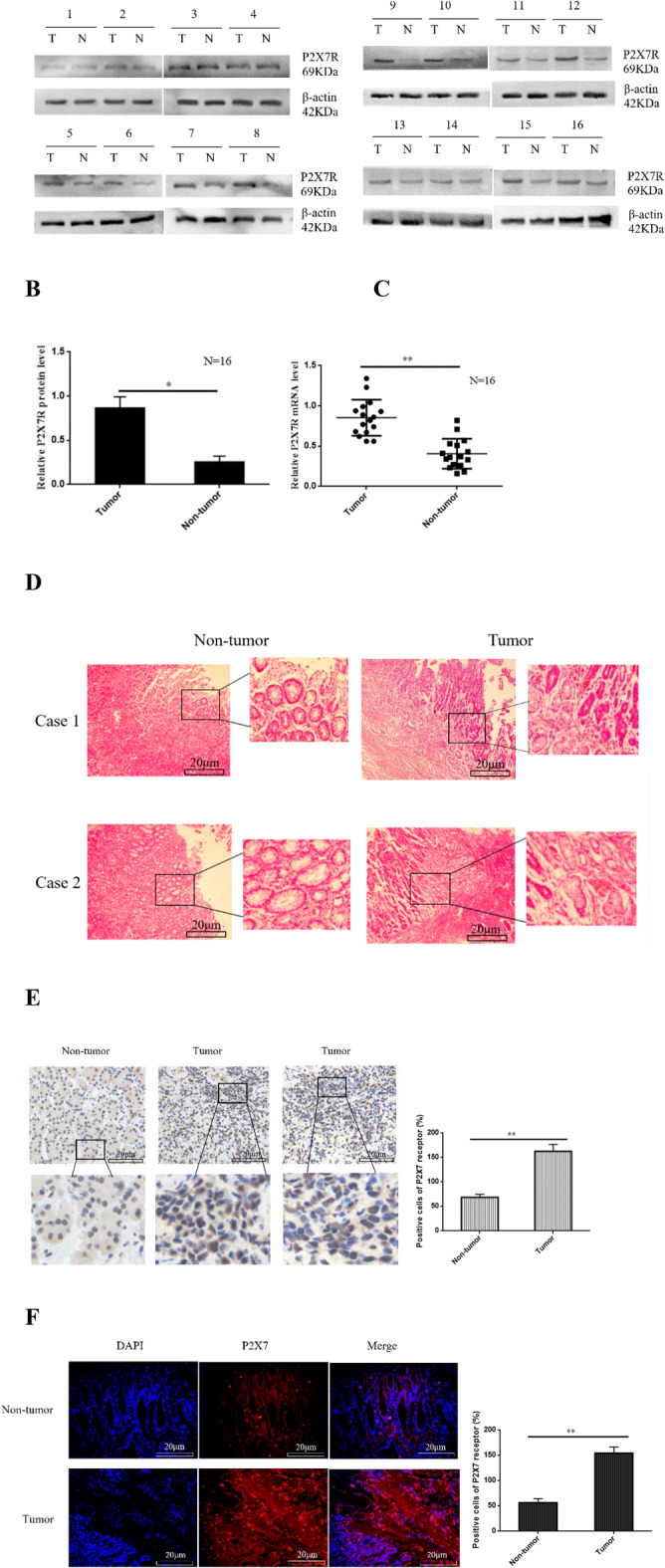
The expression of P2X7R in GC tissues. (**A**–**C**) Western-blotting and qRT-PCR were performed to measure the expression of P2X7R protein and mRNA in 16 cases of tumor and adjacent non-tumor tissues. (**D**,**E**) (Representative images): H-E staining and IHC were performed to measure the expression of P2X7R in the tumor tissues and adjacent normal tissues, bar = 50 µm. (**F**) (Representative images): Immunofluorescence was performed to detect the expression of P2X7R in the tumor tissues and adjacent non-tumor tissues, bar = 20 µm. Data are expressed as the mean ± SEM of three independent experiments. *T* tumor tissues, *N* non-tumor tissues *P < 0.05, **P < 0.01.

To investigate the expression of P2X7R in patients with GC tissues. Western-blotting and qRT-PCR were performed to detect the expression of P2X7R in 16 cases of GC tissues and adjacent non-tumor tissues. Our data showed that, compared with adjacent non-tumor tissues, the expression level of P2X7R protein and mRNA in GC tissues were significantly increased (Fig. 1A–C). Moreover, H-E staining and IHC were performed to detect the expression of P2X7R in 86 cases of GC tissues and matched adjacent non-tumor tissues. Our data showed that there were fewer P2X7R positive cells in non-tumor tissues and the staining was lighter. However, in the tumor tissues, the number of P2X7R positive cells increased significantly, and the staining was darker. (Fig. 1D,E). Furthermore, immunofluorescence results showed that the number of P2X7R labeled cells in tumor tissues was significantly higher than those in non-tumor tissues (Fig. 1F). These data indicate that P2X7R is highly expressed in GC tissues.

### High expression of P2X7R is closely related to poor survival prognosis

**Fig. 2:**
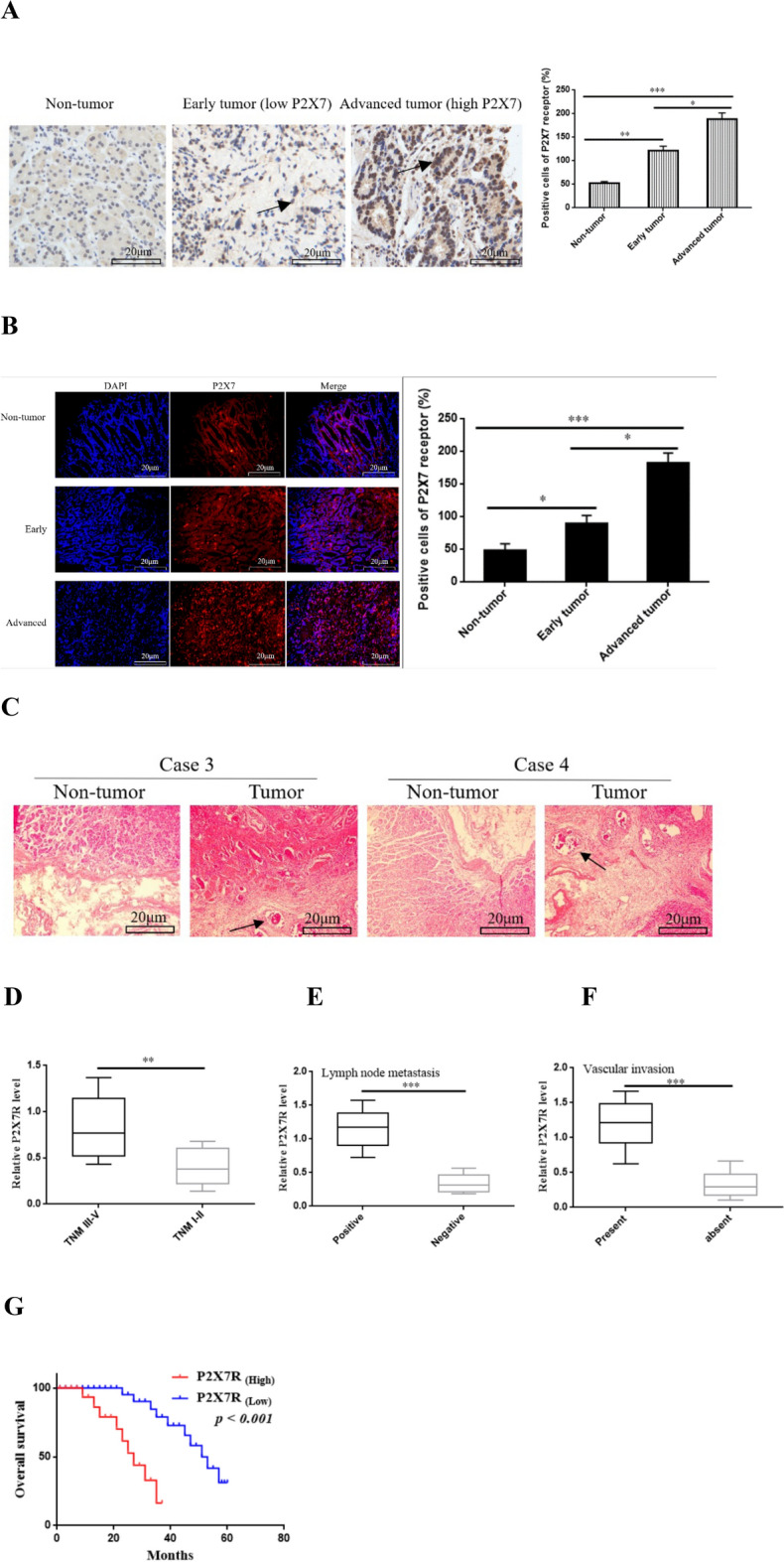
Correlation between P2X7R and clinicopathological characteristics of GC. (**A**,**B**) (Representative images): IHC and Immunofluorescence were performed to detect the expression of P2X7R in advanced GC and early GC, bar = 50 µm. (**C**–**F**) The high expression of P2X7R was closely related to TNM staging, lymph node metastasis and vascular invasion. (**G**) Kaplan–Meier survival curves indicated that high expression of P2X7R was closely related to poor overall survival prognosis. *P < 0.05, **P < 0.01, ***P < 0.001.

We investigated whether there was an intrinsic correlation between P2X7R and clinical pathological characteristics. The results were shown in Tables 1 and 2. According to the IHC-score, we divided 86 GC tissues into P2X7R high expression group (n = 46, 53.5%) and P2X7R low expression group (n = 40, 46.5%). The expression level of P2X7R in the tissues of patients with advanced GC was significantly higher than that in patients with early GC (Fig. 2A). Immunofluorescence also showed that the expression of P2X7R in the tissues of patients with advanced GC was higher than that in patients with early GC (Fig. 2B). High expression of P2X7R was closely related to TNM staging, lymph node metastasis and vascular invasion (Fig. 2C–F). However, no correlation was found between P2X7R and tumor size, age, sex and histological grade. Moreover, Kaplan–Meier survival curves indicated that high expression of P2X7R was negatively correlated with the prognosis of patients with GC (Fig. 2G). These data indicate that high expression of P2X7R is closely related to the poor overall prognosis of patients with GC.

**Table 1:** Relationship between the expression of P2X7R and the clinicopathological features of GC.

Group	Total	Expression of P2X7R		
		High	Low	*p* value
Gender				
Male	50	34 (39.53%)	16 (18.60%)	0.827
Female	36	22 (25.58%)	14 (16.28%)	
Age				
< 65	68	40 (46.51%)	28 (32.56%)	0.351
≥ 65	20	12 (13.95%)	8 (9.30%)	
Tumor site				
Proximal	22	9 (10.47%)	13 (15.12%)	0.108
Middle	16	10 (11.63%)	6 (6.98%)	
Distal	38	33 (38.57%)	5 (5.81%)	
Diffuse	10	4 (4.65%)	6 (6.98%)	
TNM stage				
I–II	24	17 (19.77%)	7 (8.14%)	0.0084**
III–IV	62	49 (56.98%)	13 (15.12%)	
Lymph node status				
Positive	68	52 (60.47%)	16 (18.60%)	0.0013**
Negative	18	6 (6.98%)	12 (13.95%)	
Vascular invasion				
Positive	54	34 (39.53%)	20 (24.56%)	0.013*
Negative	32	12 (13.95%)	20 (23.26%)	
Histological grade				
Well	3	0 (0%)	3 (3.49%)	1.103
Moderate	67	37 (43.02%)	30 (34.88%)	
Poor/undifferentiated	16	13 (15.12%)	3 (3.49%)	
Depth of tumor invasion				
T1	4	1 (1.16%)	3 (3.49%)	0.073
T2	30	14 (16.28%)	16 (18.60%)	
T3	36	28 (32.56%)	8 (9.30%)	
T4	16	11 (12.79%)	5 (5.81%)	
Tumor size				
< 3 cm	37	14 (16.28%)	23 (26.74%)	0.144
≥ 3 cm	49	19 (22.09%)	30 (34.88%)	

*p < 0.05; **p < 0.01.

**Table 2:** Univariate and multivariate cox regression analysis of the relationship between P2X7R and overall survival.

Characteristics	Univariate		Multivariate	
	HR (95%CI)	P value	HR (95%CI)	P value
Gender (male/female)	1.03 (0.68–1.57)	0.827	0.82 (0.53–1.34)	0.573
Age (≥ 60/ < 60)	1.43 (1.37–2.34)	0.072	1.03 (0.73–1.58)	0.283
Location (proximal/middle/distal/diffuse)	0.43 (0.25–0.75)	0.128	0.74 (0.76–1.17)	0.257
Lymph node metastasis (Positive/ Negative)	5.82 (3.65–7.39)	< 0.001	4.26 (2.28–8.43)	< 0.001
Tumor size (< 3 cm/≥ 3 cm)	6.52 (4.14–9.51)	< 0.001	1.94 (1.33–2.89)	0.072
TNM stage (I–II/III–IV)	3.68 (2.43–5.56)	< 0.001	2.26 (1.58–5.13)	0.003
Grade (Well/moderate/poor/undifferentiated)	1.24 (0.83–2.54)	0.178	0.83 (0.23–0.86)	0.376
Vascular invasion (absent/present)	2.14 (1.43–4.24)	< 0.001	1.02 (0.47–0.88)	0.592
Depth of tumor invasion (T1/T2/T3/T4)	2.73 (1.02–4.23)	0.042	0.64 (0.44–1.33)	0.625
P2X7R expression (high/low)	2.04 (1.26–3.85)	< 0.001	2.17 (1.36–3.69)	< 0.001

*HR* hazard ratio, *CI* confidence interval.

### P2X7R expression and functional characteristics in GC cells

**Fig. 3:**
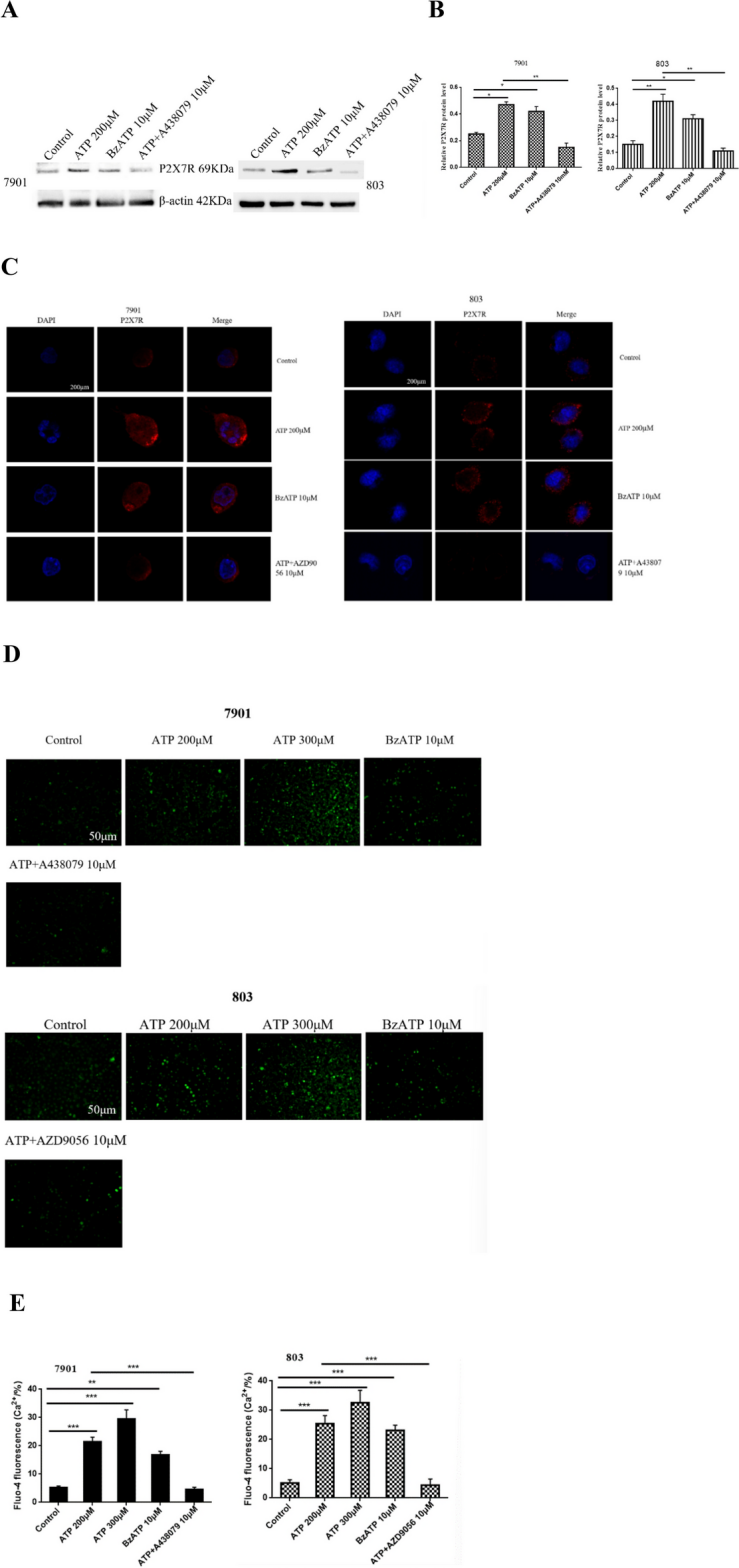
Expression and functional characteristics of P2X7R in GC cells. 7901 and 803 cells were treated or untreated with ATP (200 μM), ATP (300 μM), BzATP (10 μM), ATP + A438079 (10 μM) or ATP + AZD9056 (10 μM) for 24 h. (**A**,**B**) Western-blotting were performed to measure the expression of P2X7R in GC cells. (**C**) Immunofluorescence was used to detect the expression of P2X7R in GC cells, bar = 200 μm. (**D**,**E**) Fluo-4AM fluorescence technology was performed to detect the intracellular calcium concentration of GC cells, bar = 50 μm. Data are expressed as the mean ± SEM of three independent experiments. *P < 0.05, **P < 0.01, *** P < 0.001.

To investigate the expression of P2X7R in GC cells. 7901 and 803 cells were treated or untreated with ATP (200 μM), BzATP (10 μM), or ATP + A438079 (10 μM) for 24 h. Western-blotting showed that ATP and BzATP increased the expression of P2X7R in GC cells, while A438079 decreased the expression of P2X7R (Fig. 3A,B). Moreover, we detected the expression of P2X7R in GC cells by immunofluorescence, data showed that P2X7R was mainly expressed on the membrane of GC cells. ATP and BzATP significantly increased the fluorescence intensity of P2X7R labeled cells. While A438079 inhibited the changes induced by ATP (Fig. 3C).

Additionally, activation of P2X7R can open the cation channel on the cell membrane and induce calcium ion influx. To understand the functional characteristics of P2X7R in GC cells. Fluo-4AM fluorescence technology was performed to detect intracellular calcium concentration in GC cells. 7901 and 803 cells were treated or untreated with ATP (200 μM), ATP (300 μM), BzATP (10 μM), ATP + A438079 (10 μM) or ATP + AZD9056 (10 μM) for 30 min. Our data showed that ATP and BzATP increased the intracellular calcium concentration, while A438079 and AZD9056 inhibited these ATP-induced changes (Fig. 3D,E). These data indicate that P2X7R is a membrane receptor and expresses in GC cells, indicating that P2X7R may play a certain function in GC cells.

### Activation of P2X7R promotes the proliferation of GC cells

**Fig. 4:**
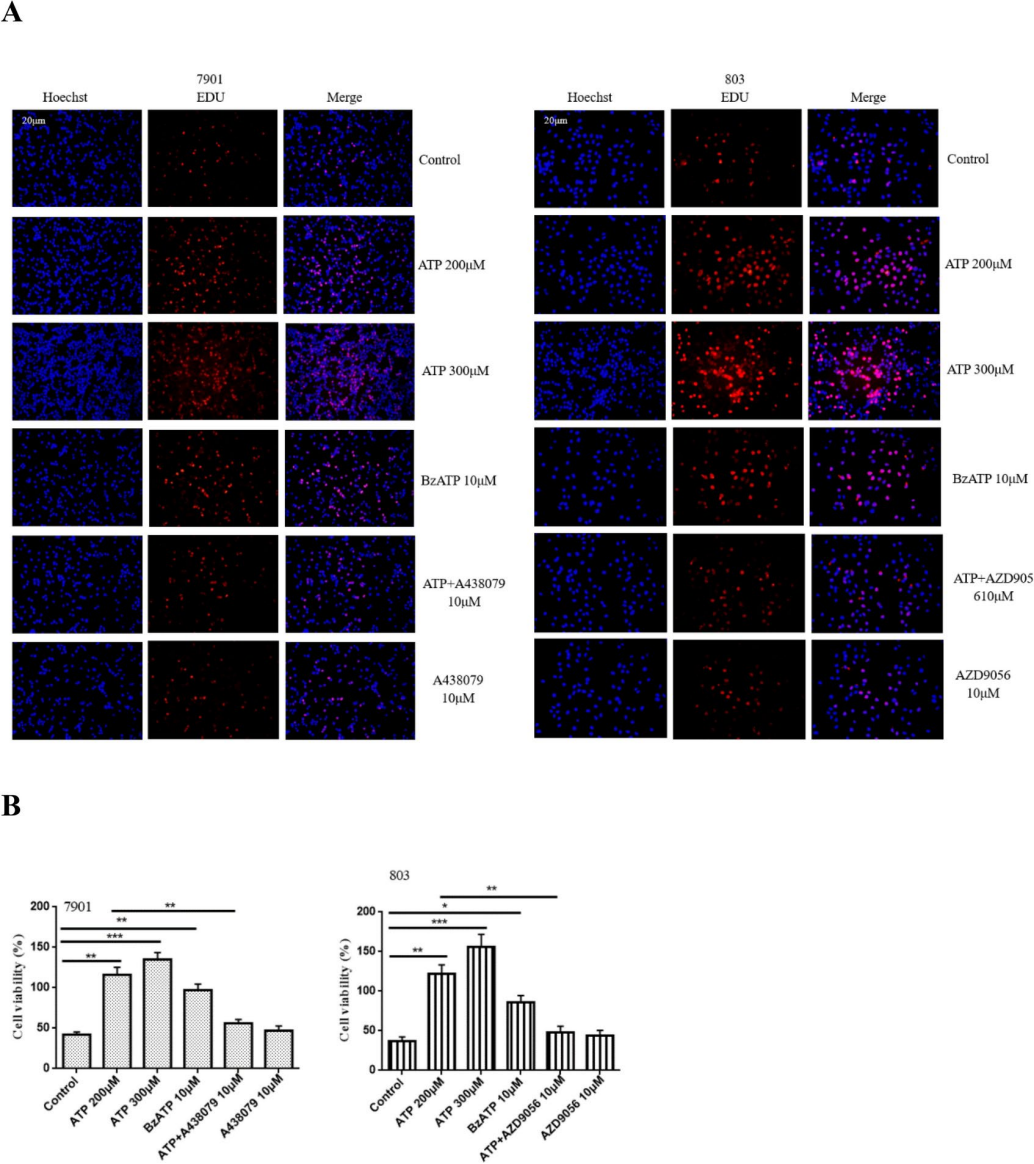
The effect of P2X7R on the activities of GC cells. 7901 and 803 cells were treated with ATP (200 μM), ATP (300 μM), BzATP (10 μM), A438079 (10 μM), ATP + A438079 (10 μM), AZD9056 (10 μM) or ATP + AZD9056 (10 μM) for 24 h. (**A**,**B**) EDU assay was performed to detect the proliferation abilities of GC cells, bar = 20 μm. Data are expressed as the mean ± SEM of three independent experiments. *P < 0.05, **P < 0.01, ***P < 0.001.

To investigate the effect of P2X7R on the changes in the proliferation of GC cells. 7901 and 803 cells were treated or untreated with ATP (200 μM), ATP (300 μM), BzATP (10 μM), A438079 (10 μM), ATP + A438079 (10 μM), AZD9056 (10 μM) or ATP + AZD9056 (10 μM) for 24 h. Figure 4A,B showed that ATP and BzATP increased the proliferation activities of GC cells, while A438079 inhibited the abilities of GC cells induced by ATP. However, in the absence of ATP, P2X7R antagonist alone had no significant effect on reducing the proliferation. This indicates that the activation of P2X7R can promote the proliferation of GC cells.

### P2X7R overexpression promotes the migration and invasion of GC cells

**Fig. 5:**
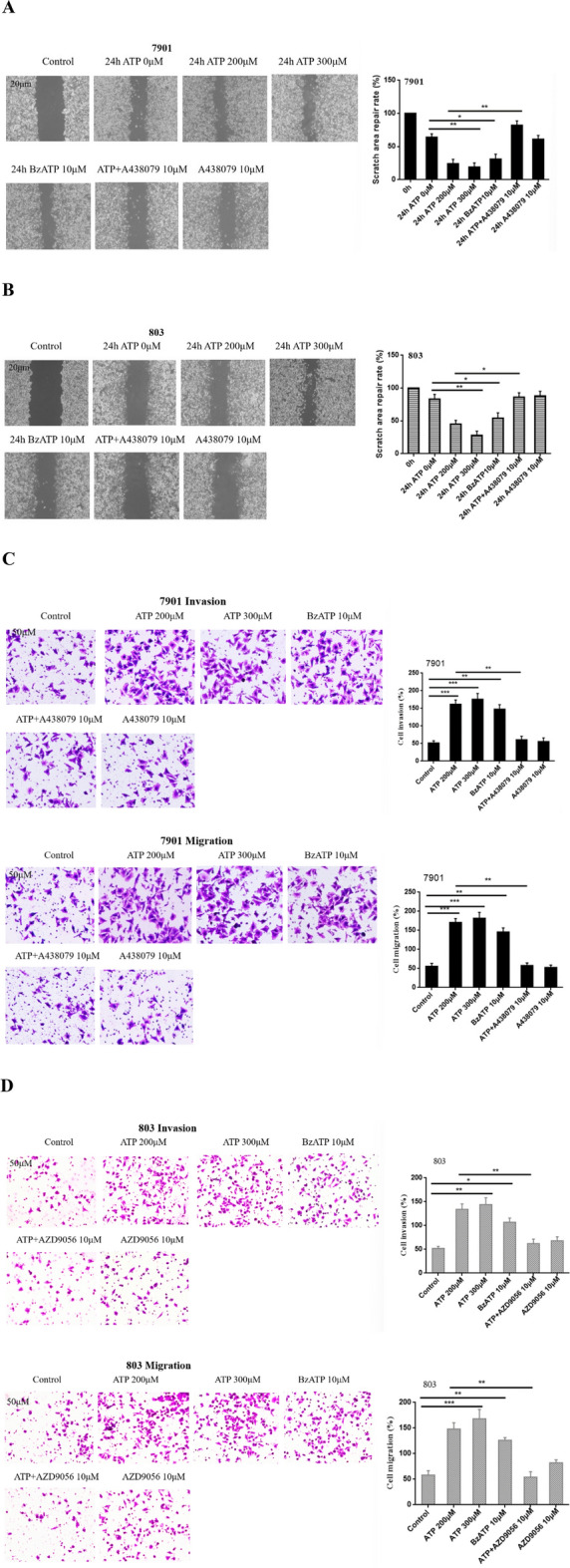
Effect of P2X7R on migration and invasion of GC cells. 7901 and 803 cells were treated with ATP (200 μM), ATP (300 μM), BzATP (10 μM), A438079 (10 μM), ATP + A438079 (10 μM), AZD9056 (10 μM), or ATP + AZD9056 (10 μM) for 24 h. (**A**,**B**) Cell scratch assay was performed to detect the migration abilities of P2X7R in GC cells, bar = 20 μm. (**C**,**D**) Transwell migration and invasion assays were performed to detect the migration and invasion abilities of P2X7R on GC cells, bar = 50 μm. Data are expressed as the mean ± SEM of three independent experiments. * P < 0.05, **P < 0.01, ***P < 0.001.

To investigate the effect of P2X7R on the migration and invasion abilities of GC cells. 7901 and 803 cells were treated or untreated with ATP (200 μM), ATP (300 μM), BzATP (10 μM), A438079 (10 μM), ATP + A438079 (10 μM), AZD9056 (10 μM) or ATP + AZD9056 (10 μM) for 24 h. Cell scratch results found that ATP and BzATP promoted the migration of GC cells. While A438079 inhibited the migration of GC cells induced by ATP. However, in the absence of ATP, the inhibitory effect of the antagonist on the migration of GC cells was not obvious (Fig. 5A,B).

Moreover, Transwell invasion and migration assays were performed to detect the invasion and migration abilities of GC cells. Our data showed that ATP and BzATP significantly promoted the invasion and migration abilities of GC cells. While the use of A438079 and AZD9056 inhibited the invasion and migration of GC cells induced by ATP. However, in the absence of ATP, the inhibitory effect of the inhibitors was not obvious (Fig. 5C,D). These data show that P2X7R activation plays a significant role in promoting the motor abilities of GC cells.

### Activation of P2X7R enhances the stress ability of actin fibers

**Fig. 6:**
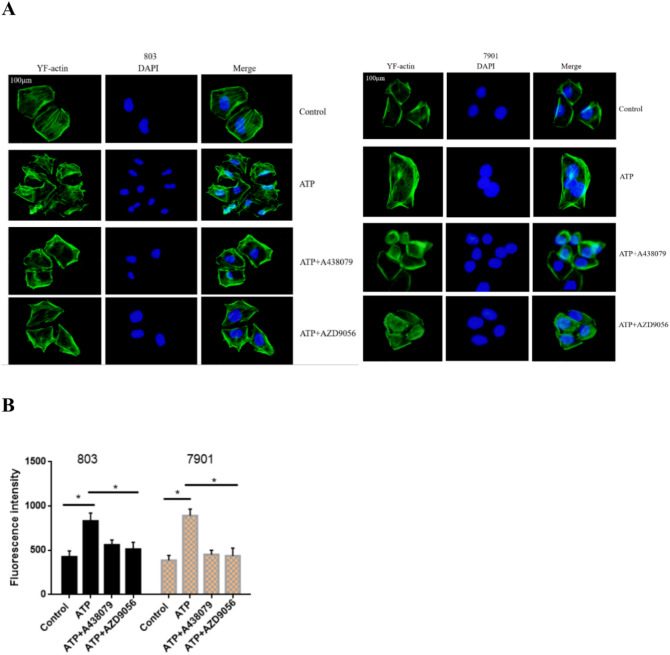
The effect of P2X7R on actin fibers of GC cells. (**A**,**B**) Actin fiber staining assay was carried out to detect the actin fiber changes of GC cells, bar = 100 μm, *P < 0.05.

The movement and migration of cells require the participation of actin fibers, and the enhancement of actin fibers can cause cells to move quickly^21–23^. Therefore, we detected stress changes in the actin fibers of GC cells through actin staining assay. 803 and 7901 cells were treated with ATP (200 μM), ATP + A438079 (10 μM) and ATP + AZD9056 (10 μM) for 24 h. The results found that, compared with the control group, the actin stress response in the 803 and 7901 cells of the ATP treatment group was significantly enhanced, the actin filaments and networks were enhanced, the number of protruding pseudopods at the edges increased, and the cell morphology changed significantly. Conversely, the application of A438079 and AZD9056 reversed this phenomenon (Fig. 6A,B). These data show that the activation of P2X7R promote the motilities of GC cells.

## Discussion

Early detection and interventional therapy of GC are the key to cure GC, which depends on the exploration of key molecules conducive to the prediction of GC. Therefore, it is of great significance to explore the molecular basis related to the pathogenesis of GC and to find effective anticancer targets. Different studies have revealed that P2X7R plays an important regulatory role in tumor progression and can be used as predictive molecular targets for tumors, such as colorectal cancer^24^, lung cancer^25^ and bladder cancer^6^. The expression of P2X7R in these tumors showed a trend of high expression, which was negatively correlated with the prognosis of patients. Studies have shown that patients with colorectal cancer have high P2X7R or low P2X7R population. Patients with the high expression of P2X7R have relatively short survival time, higher serum carcinoembryonic antigen levels and more advanced tumors^24^. Univariate and multivariate COX regression analysis showed that the high expression of P2X7R could be used as an independent prognostic factor affecting the overall survival rate of patients with colorectal cancer^26^. In this study, we also found that P2X7R was highly expressed in GC tissues, and its high expression was closely related to lymph node metastasis, vascular invasion and TNM. The high expression of P2X7R was negatively correlated with the overall survival and prognosis of patients, which suggests that P2X7R can be used as a favorable biological marker for the prediction of GC.

Generally speaking, the growth, migration and metastasis of tumor cells do not depend on themselves, but on the microenvironment in which they live. Tumor cells can adapt to their own growth by changing this microenvironment. Tumor microenvironment includes many substances, such as ATP. ATP can be released by tumor cells and non-tumor cells in microenvironment. These released ATP involved in regulating the biological behavior of tumor cells through metabolism and binding to related ligands^13,27,28^. The increase of ATP level in tumor microenvironment leads to the increase of COX-2 expression, which in turn provides the characteristics of migration and invasion for tumors^29^. Studies have shown that ATP promote the invasion and drug resistance of breast cancer cells. After ATP treatment of breast cancer cells, the expression of Y-box 9 (SOX9) in sex determination area was up-regulated. While the SOX9 gene was knocked out, the invasiveness of cells decreased^30^. SOX9 gene knockout and apyrase (an ATP hydrolase) treatment of MDA-MB-231 cells showed inhibition of tumor growth and increase of drug sensitivity in nude mice^30^. ATP can stimulate the signal transduction of HIF and upregulate the expression of hypoxia inducible factor 1 up 2α (HIF-1/2α). After knocking out HIF-1/2α with siRNA, it was found that HIF1/2α-siRNA significantly reduced the invasion and EMT of breast cancer cells driven by ATP, suggesting that ATP may promote the invasion and metastasis of breast cancer through HIF-2α signal^31^. In this study, we found that ATP and its analogue BzATP significantly increased the calcium concentration of GC cells and promoted the growth, migration and invasion of GC cells. These experimental results are consistent with the previous studies, suggesting the regulatory effect of ATP on GC.

Different studies have confirmed that P2X7R is highly expressed in most tumor cells. As a natural activator of P2X7R, ATP participates in the regulation of tumor cell progression by activating P2X7R. P2X7R activation mediates the opening of ion channels on the membrane (calcium and sodium ion influx, potassium ion efflux), which further activates intracellular signals and regulates intracellular molecular metabolism, thus affecting the activity of tumor cells^32,33^. P2X7R in highly invasive breast cancer cells induces cell morphological changes through rapid F-actin recombination and filamentous pseudopodia formation, and promotes the invasion of tumor cells through two-dimensional and three-dimensional extracellular matrix in vitro^34^. The use of exogenous agonists (ATP and BzATP) to act on P2X7R promotes the growth and migration of tumor cells^33,35^. ATP and BzATP induce the activation of P2X7R and promote the proliferation, migration and invasion of colorectal cancer cells. On the contrary, antagonizing the activity of P2X7R can inhibit the proliferation and migration of colorectal cancer cells^18^. It was further found that P2X7R antagonist could inhibit the migration of colorectal cancer cells induced by TGF-β1^18^. P2X7R antagonist A438079 can inhibit the proliferation, invasion and migration of HCT-116 and SW620 cells, and inhibit the growth of colorectal cancer xenografts in nude mice^36^. In patient-derived primary glioblastoma cultures and U251 human glioblastoma cell lines, P2X7R activation promotes tumor progression, while inhibition of its activity reduces tumor growth and depletes the number of glioblastoma cells in vitro^37^. Other studies have shown that P2X7R activation promotes neuroblastoma growth and proliferation by activating PI3K/GSK3 β/VEGF signaling, while the use of P2X7R antagonists (AZ10606120 or A740003) reduces neuroblastoma derived tumor growth^38^. In addition, tumor growth and metastatic spread are severely affected by P2X7R and microbubbles and exosomes released into the tumor microenvironment. Studies have shown that P2X7R stimulation triggers the release of miRNA-containing microvesicles and exosomes in melanoma cells. These miRNAs promote the growth and migration of melanoma cells^9^. These studies have revealed that P2X7R is an important regulatory molecule of tumor. However, little is known about the effects of P2X7R on the proliferation, migration and invasion of GC cells. In this study, we found that P2X7R was highly expressed in gastric cancer cell lines. Activation of P2X7R by ATP and its analogue BzATP promoted the proliferation, migration and invasion of GC cells. Activation of P2X7R promoted YF actin stress and enhanced the motor abilities of GC cells. While the use of P2X7R antagonist A438079 and AZD9056 inhibited these changes in GC cells induced by ATP.

In short, the data obtained in this experiment suggest that P2X7R may be used as a favorable molecular target for GC. P2X7R activation can promote the proliferation, migration and invasion of GC cells, but antagonizing the activity of P2X7R produces the opposite result. These data provide a basis for further investigation of the molecular mechanism and functional role of P2X7R in regulating the progression of GC.

## Supplementary Information

Supplementary Information.

## Data Availability

All data generated or analyzed during this study are included in this article. And we have not used other data that has already been published. All the data presented in this article are original results derived from this study.

## References

[1] Zou, F. L., Liu, J. P., Zuo, C., He, P. F., Ye, J. X. & Zhang, W. J. The functional role of P2 purinergic receptors in the progression of gastric cancer. *Purinergic Signal*. (2024). 10.1007/s11302-024-10000-7 PMC12454698 38470513

[2] Smyth, E. C., Nilsson, M., Grabsch, H. I., van Grieken, N. C. & Lordick, F. Gastric cancer. *Lancet* **396**(10251), 635–648 (2020). 32861308 10.1016/S0140-6736(20)31288-5

[3] Rabelo, I. L. A., Arnaud-Sampaio, V. F., Adinolfi, E., Ulrich, H. & Lameu, C. Cancer metabostemness and metabolic reprogramming via P2X7 receptor. *Cells* **10**(7), 1782 (2021). 34359950 10.3390/cells10071782 PMC8305434

[4] Hu, S. Q. et al. P2X7 receptor in inflammation and pain. *Brain Res. Bull.* **187**, 199–209 (2022). 35850190 10.1016/j.brainresbull.2022.07.006

[5] Matyśniak, D. et al. P2X7 receptor: the regulator of glioma tumor development and survival. *Purinergic Signal.* **18**(1), 135–154 (2022). 34964926 10.1007/s11302-021-09834-2 PMC8850512

[6] Ledderose, S. et al. P2X1 and P2X7 receptor overexpression is a negative predictor of survival in muscle-invasive bladder cancer. *Cancers (Basel)* **15**(8), 2321 (2023). 37190249 10.3390/cancers15082321 PMC10136747

[7] de Araújo, J. B., Kerkhoff, V. V., de Oliveira Maciel, S. F. V., de Resende E Silva, D. T. Targeting the purinergic pathway in breast cancer and its therapeutic applications. *Purinergic Signal*. **17**(2), 179–200 (2021). 10.1007/s11302-020-09760-9 PMC7879595 33576905

[8] Zhang, W. J., Zhang, L. P., Lin, S. J., Wang, C. Y., Le, Y. G. P2 purinergic receptors regulate the progression of colorectal cancer. *Purinergic Signal*. (2023). 10.1007/s11302-023-09983-6 PMC12454802 38153612

[9] Pegoraro, A. et al. P2X7 promotes metastatic spreading and triggers release of miRNA-containing exosomes and microvesicles from melanoma cells. *Cell Death Dis.* **12**(12), 1088 (2021). 34789738 10.1038/s41419-021-04378-0 PMC8599616

[10] Pegoraro, A. et al. Differential sensitivity of acute myeloid leukemia cells to daunorubicin depends on P2X7A versus P2X7B receptor expression. *Cell Death Dis.* **11**(10), 876 (2020). 33071281 10.1038/s41419-020-03058-9 PMC7569086

[11] Giuliani, A. L. et al. Trophic activity of human P2X7 receptor isoforms A and B in osteosarcoma. *PLoS One* **9**(9), e107224 (2014). 25226385 10.1371/journal.pone.0107224 PMC4165768

[12] Adinolfi, E., Amoroso, F. & Giuliani, A. L. P2X7 receptor function in bone-related cancer. *J. Osteoporos.* **2012**, 637863 (2012). 22970409 10.1155/2012/637863 PMC3431089

[13] Di Virgilio, F., Vultaggio-Poma, V. & Sarti, A. C. P2X receptors in cancer growth and progression. *Biochem. Pharmacol.* **187**, 114350 (2021). 33253643 10.1016/j.bcp.2020.114350

[14] Zuo, C., Xu, Y. S., He, P. F. & Zhang, W. J. ATP ion channel P2X7 receptor as a regulatory molecule in the progression of colorectal cancer. *Eur. J. Med. Chem.* **261**, 115877 (2023). 37857146 10.1016/j.ejmech.2023.115877

[15] De Marchi, E. et al. The P2X7 receptor modulates immune cells infiltration, ectonucleotidases expression and extracellular ATP levels in the tumor microenvironment. *Oncogene* **38**(19), 3636–3650 (2019). 30655604 10.1038/s41388-019-0684-y PMC6756114

[16] Kan, L. K. et al. P2X7 receptor antagonism inhibits tumour growth in human high-grade gliomas. *Purinergic Signal.* **16**(3), 327–336 (2020). 32583309 10.1007/s11302-020-09705-2 PMC7524927

[17] Bai, X. et al. P2X7 receptor promotes migration and invasion of non-small cell lung cancer A549 cells through the PI3K/Akt pathways. *Purinergic Signal.* **19**(4), 685–697 (2023). 36854856 10.1007/s11302-023-09928-z PMC10754800

[18] Zhang, W. J. et al. PI3K/Akt/GSK-3β signal pathway is involved in P2X7 receptor-induced proliferation and EMT of colorectal cancer cells. *Eur. J. Pharmacol.* **899**, 174041 (2021). 33737010 10.1016/j.ejphar.2021.174041

[19] Jelassi, B. et al. P2X(7) receptor activation enhances SK3 channels- and cystein cathepsin-dependent cancer cells invasiveness. *Oncogene* **30**(18), 2108–2122 (2011). 21242969 10.1038/onc.2010.593

[20] Adinolfi, E. et al. Expression of P2X7 receptor increases in vivo tumor growth. *Cancer Res.* **72**(12), 2957–2969 (2012). 22505653 10.1158/0008-5472.CAN-11-1947

[21] Begum, R., Nur-E-Kamal, M. S. & Zaman, M. A. The role of Rho GTPases in the regulation of the rearrangement of actin cytoskeleton and cell movement. *Exp. Mol. Med.* **36**, 358–366 (2004). 15365255 10.1038/emm.2004.47

[22] Janota, C. S., Calero-Cuenca, F. J. & Gomes, E. R. Methods to measure perinuclear actin dynamics during nuclear movement in migrating cells. *Methods Mol. Biol.* **2101**, 371–385 (2020). 31879914 10.1007/978-1-0716-0219-5_21

[23] Blanchoin, L., Boujemaa-Paterski, R., Sykes, C. & Plastino, J. Actin dynamics, architecture, and mechanics in cell motility. *Physiol. Rev.*, 235–263 (2014). 10.1152/physrev.00018.2013 24382887

[24] Zhang, Y., Ding, J. & Wang, L. The role of P2X7 receptor in prognosis and metastasis of colorectal cancer. *Adv. Med. Sci.* **64**(2), 388–394 (2019). 31276917 10.1016/j.advms.2019.05.002

[25] Li, Q., Zhu, X., Song, W., Peng, X. & Zhao, R. The P2X7 purinergic receptor: a potential therapeutic target for lung cancer. *J. Cancer Res. Clin. Oncol.* **146**(11), 2731–2741 (2020). 32892231 10.1007/s00432-020-03379-4 PMC11804286

[26] Qian, F. et al. High expression of P2X7R is an independent postoperative indicator of poor prognosis in colorectal cancer. *Hum. Pathol.* **64**, 61–68 (2017). 28412208 10.1016/j.humpath.2017.03.019

[27] Kepp, O. et al. ATP and cancer immunosurveillance. *EMBO J.* **40**(13), e108130 (2021). 34121201 10.15252/embj.2021108130 PMC8246257

[28] Chiarella, A. M., Ryu, Y. K., Manji, G. A. & Rustgi, A. K. Extracellular ATP and adenosine in cancer pathogenesis and treatment. *Trends Cancer* **7**(8), 731–750 (2021). 34074623 10.1016/j.trecan.2021.04.008

[29] Sharma, S., Kalra, H. & Akundi, R. S. Extracellular ATP mediates cancer cell migration and invasion through increased expression of cyclooxygenase 2. *Front. Pharmacol.* **11**, 617211 (2021). 33584298 10.3389/fphar.2020.617211 PMC7873692

[30] Yang, H. et al. Extracellular ATP promotes breast cancer invasion and chemoresistance via SOX9 signaling. *Oncogene* **39**(35), 5795–5810 (2020). 32724162 10.1038/s41388-020-01402-z

[31] Magni, L. et al. The P2X7 receptor stimulates IL-6 release from pancreatic stellate cells and tocilizumab prevents activation of STAT3 in pancreatic cancer cells. *Cells* **10**(8), 1928 (2021). 34440697 10.3390/cells10081928 PMC8391419

[32] Zhang, G. P. et al. Ion channel P2X7 receptor in the progression of cancer. *Front. Oncol.* **13**, 1297775 (2024). 38273855 10.3389/fonc.2023.1297775 PMC10808724

[33] Brisson, L. et al. P2X7 receptor promotes mouse mammary cancer cell invasiveness and tumour progression, and is a target for anticancer treatment. *Cancers (Basel)* **12**(9), 2342 (2020). 32825056 10.3390/cancers12092342 PMC7565976

[34] Yang, H. et al. Extracellular ATP promotes breast cancer invasion and epithelial-mesenchymal transition via hypoxia-inducible factor 2α signaling. *Cancer Sci.* **110**(8), 2456–2470 (2019). 31148343 10.1111/cas.14086 PMC6676128

[35] Jelassi, B. et al. Anthraquinone emodin inhibits human cancer cell invasiveness by antagonizing P2X7 receptors. *Carcinogenesis* **34**(7), 1487–1496 (2013). 23524196 10.1093/carcin/bgt099

[36] Zhang, Y., Li, F., Wang, L. & Lou, Y. A438079 affects colorectal cancer cell proliferation, migration, apoptosis, and pyroptosis by inhibiting the P2X7 receptor. *Biochem. Biophys. Res. Commun.* **558**, 147–153 (2021). 33915328 10.1016/j.bbrc.2021.04.076

[37] Kan, L. K. et al. P2X7 receptor antagonism by AZ10606120 significantly reduced in vitro tumour growth in human glioblastoma. *Sci. Rep.* **13**(1), 8435 (2023). 37225786 10.1038/s41598-023-35712-5 PMC10209214

[38] Amoroso, F. et al. The P2X7 receptor is a key modulator of the PI3K/GSK3β/VEGF signaling network: evidence in experimental neuroblastoma. *Oncogene* **34**(41), 5240–5251 (2015). 25619831 10.1038/onc.2014.444

