# Supplementary figures and images for: Role of P2X7 receptor in the progression and clinicopathological characteristics of gastric cancer
Source: Sci Rep. 2024 Dec 30;14:31673. doi: 10.1038/s41598-024-81515-7 (PMC11685580; doi:10.1038/s41598-024-81515-7)

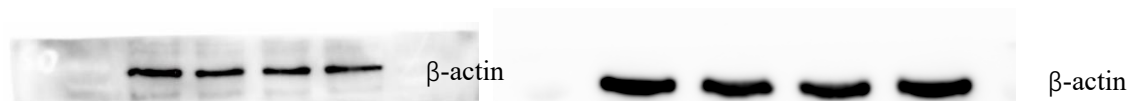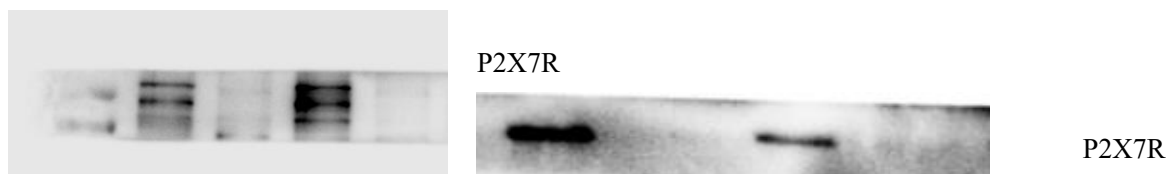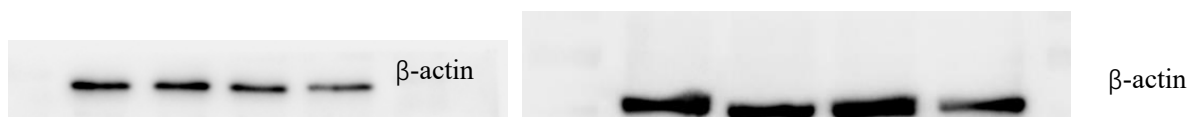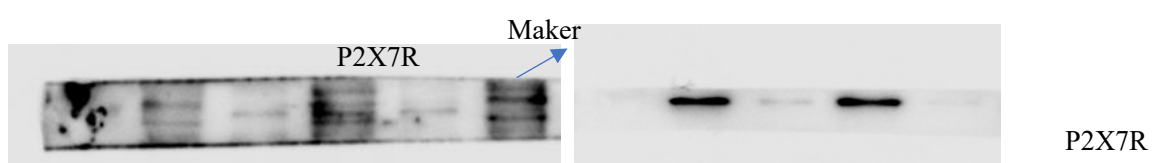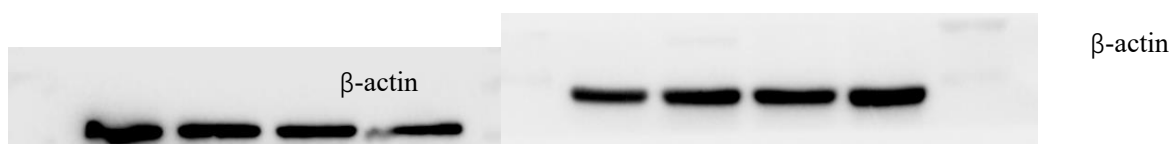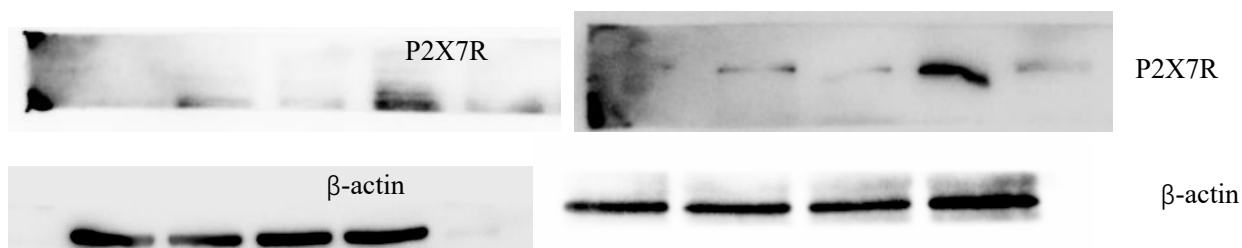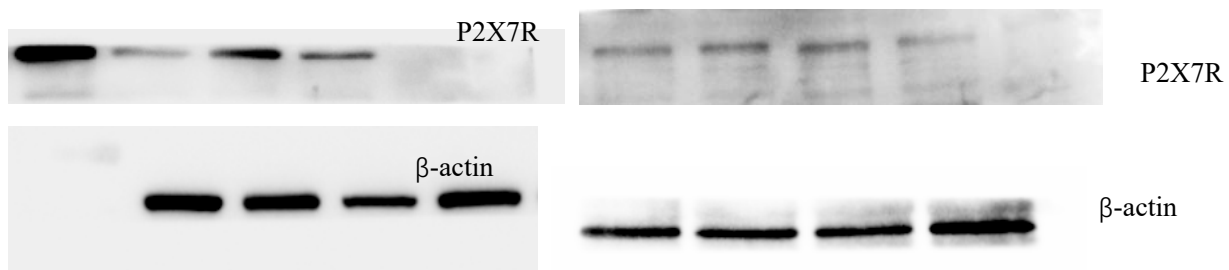

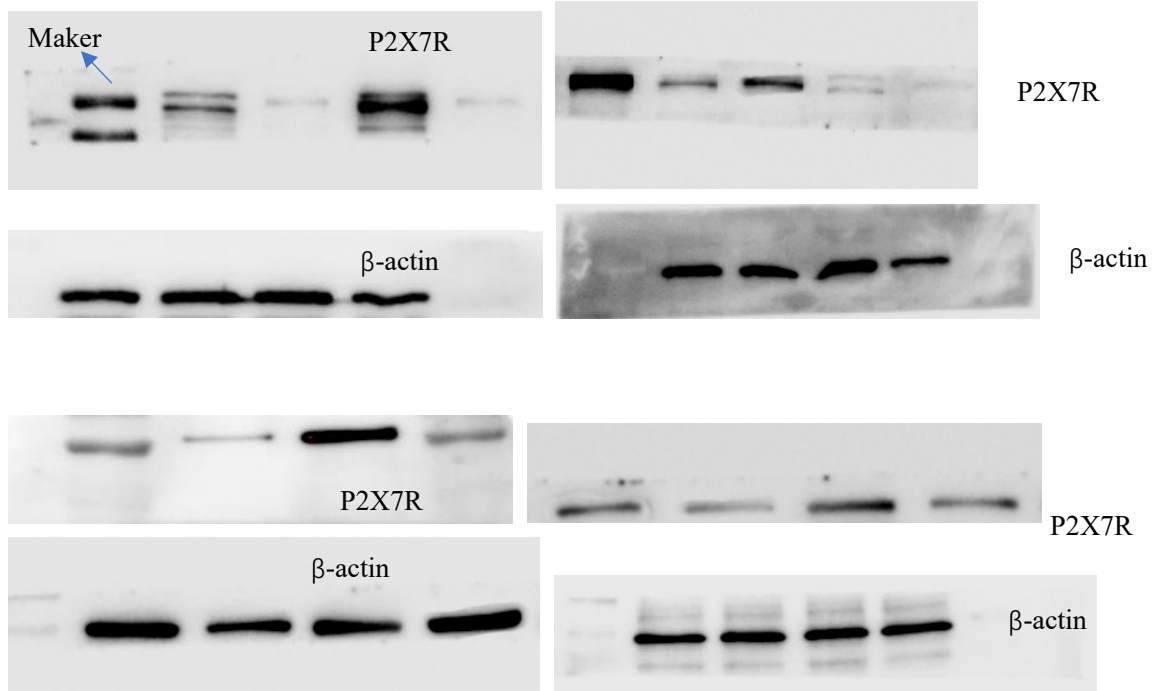

Fig 3A

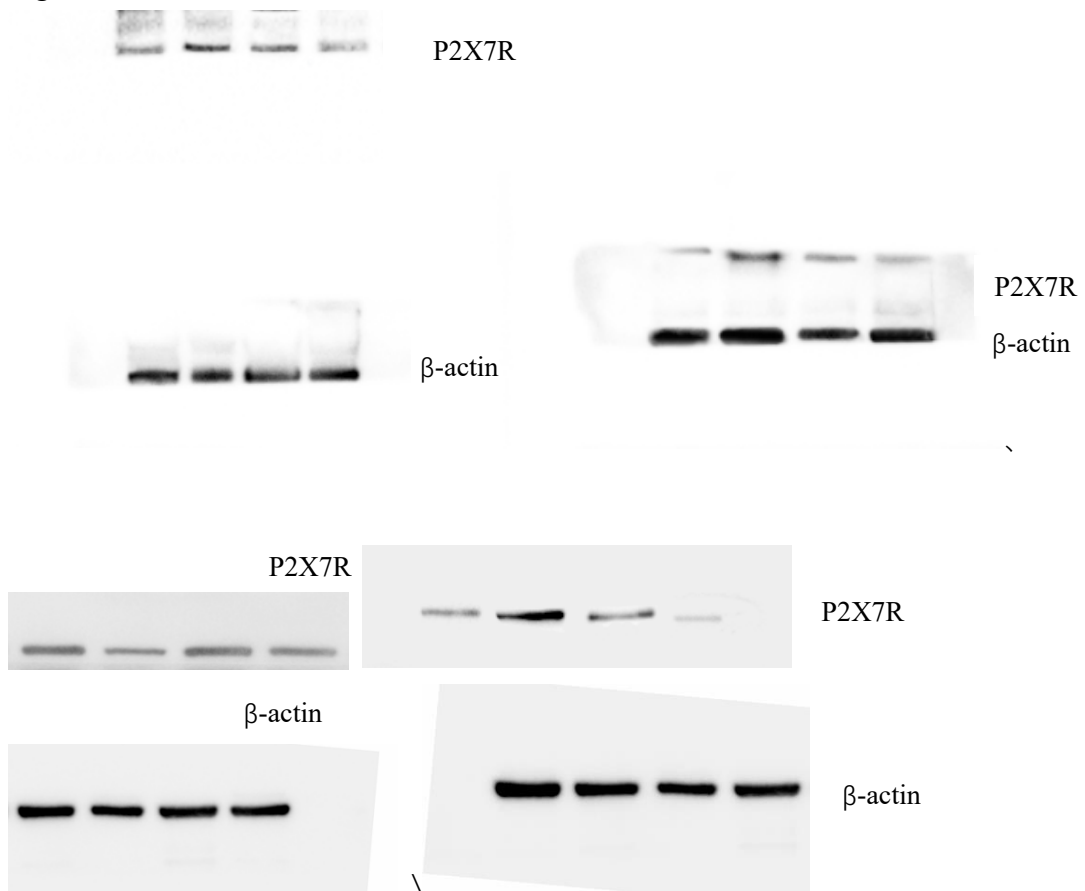

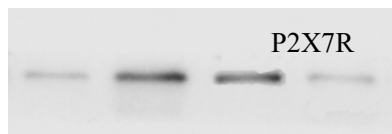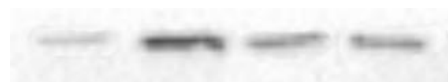

P2X7R

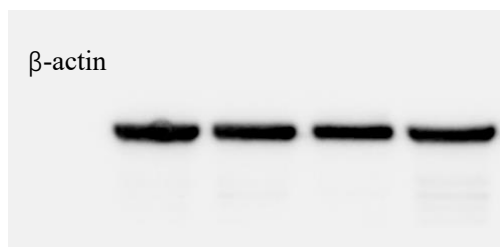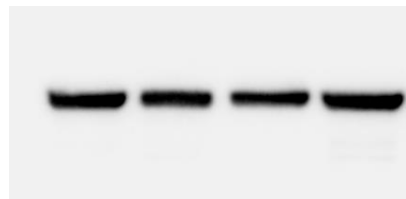

β-actin

Supplement: Supplementary file 1 — Supplementary Information. [file 41598_2024_81515_MOESM1_ESM.pdf]
